# The Autonomic Imbalance of Myocardial Ischemia during Exercise Stress Testing: Insight from Short-Term Heart Rate Variability Analysis

**DOI:** 10.3390/ijerph192215096

**Published:** 2022-11-16

**Authors:** Ping-Yen Lin, Cheng-Ting Tsai, Chang Francis Hsu, Ying-Hsiang Lee, Han-Ping Huang, Chun-Che Huang, Lawrence Yu-Min Liu, Long Hsu, Ten-Fang Yang, Po-Lin Lin

**Affiliations:** 1 Department of Electrophyics, National Yang Ming Chiao Tung University, Hsinchu 30010, Taiwan; 2 Cardiovascular Center, MacKay Memorial Hospital, Taipei 104217, Taiwan; 3 Department of Cosmetic Applications and Management, MacKay Junior College of Medicine, Nursing and Management, Taipei 25245, Taiwan; 4 Labfront (Kiipo Co.), Boston, MA 02067, USA; 5 Department of Medicine, MacKay Medical College, New Taipei City 25245, Taiwan; 6 Department of Artificial Intelligence and Medical Application, MacKay Junior College of Medicine, Nursing and Management, Taipei 25245, Taiwan; 7 Department of Healthcare Administration, I-Shou University, Kaohsiung 82445, Taiwan; 8 Division of Cardiology, Hsinchu MacKay Memorial Hospital, Hsinchu 30071, Taiwan; 9 Department of Biological Science and Technology, National Yang Ming Chiao Tung University, Hsinchu 30010, Taiwan; 10 Graduate Institute of Medical Informatics, Taipei Medical University Hospital, Taipei 110301, Taiwan

**Keywords:** exercise stress testing, coronary artery disease, myocardial ischemia, heart rate variability, short-term HRV

## Abstract

Exercise stress testing (EST) has limited power in diagnosing obstructive coronary artery disease (CAD). The heart rate variability (HRV) analysis might increase the sensitivity of CAD detection. This study aimed to evaluate the correlation between short-term HRV and myocardial ischemia during EST, including the acceleration, maximum, and recovery stages of heart rate (HR). The HRV during EST from 19 healthy (RHC) subjects and 35 patients with CAD (25 patients with insignificant CAD (iCAD), and 10 patients with significant CAD (sCAD)) were compared. As a result, all HRV indices decreased at the maximum stage and no significant differences between iCAD and sCAD were found. The low-frequency power of heart rate signal (LF) of the RHC group recovered relatively quickly from the third to the sixth minutes after maximum HR, compared with that of the sCAD group. The relative changes of most HRV indices between maximum HR and recovery stage were lower in the sCAD group than in the RHC group, especially in LF, the standard deviation of all normal to normal intervals (SDNN), and the standard deviation in the long axis direction of the Poincaré plot analysis (SD2) indices (*p* < 0.05). The recovery slope of LF was significantly smaller in the sCAD group than in the RHC group (*p* = 0.02). The result suggests that monitoring short-term HRV during EST provides helpful insight into the cardiovascular autonomic imbalance in patients with significant CAD. The relative change of autonomic tone, especially the delayed sympathetic recovery, could be an additional marker for diagnosing myocardial ischemia.

## 1. Introduction

Coronary artery disease (CAD), a narrowing or obstruction of coronary arteries, is the world’s most common cause of death [1]. Therefore, early diagnosis of CAD plays an important role in the primary prevention of cardiac death [1]. CAD leads to abnormal electrocardiograms (ECGs) and corresponding arterial waveforms. The abnormal features are enhanced for a CAD patient in exercise. The effects of exercise on the heart can be monitored via an ECG for a subject running on a treadmill in exercise stress testing (EST). Thus, EST is commonly used as a clinical approach to diagnosing CAD [2]. However, the diagnostic accuracy of EST can be influenced by the patient’s age, sex, or clinical characteristics [3]. EST has limited power to rule in or rule out obstructive CAD compared with diagnostic imaging tests [4]. In practice, EST has a low sensitivity of 68% and specificity of 77% for the detection of CAD [5]. The positive results of EST should be treated with caution because of its high false positive rate. According to the 2019 European Society of Cardiology (ESC) Guidelines for diagnosing and managing chronic coronary syndromes, EST is only recommended for risk assessment rather than for a diagnosis of CAD [4]. To clarify the diagnosis of CAD, additional coronary anatomic imaging or computed tomography (CT) is required [3], which is time-consuming or expensive. Therefore, optimizing the accuracy of EST would reduce the financial burden and medical resources.

On the other hand, heart rate variability (HRV) is a relevant marker reflecting the cardiac variation by the sympathetic and vagal components of the autonomic nervous system. Autonomic dysfunction directly correlates with the morbidity and mortality caused by CAD and involves the development of cardiovascular disease or the progression of metabolic disease [6,7]. For example, vagal-mediated HRV indices can be applied for recognizing healthy and diseased states, and have been inversely associated with metabolic diseases, including diabetes, central obesity, dyslipidemia, and hypertension [7]. In the vagal-mediated HRV analysis, long-term HRV was largely applied to predict sudden cardiac death [7]. Short-term HRV is also a method used for enhanced risk assessment in low- to intermediate-risk individuals without known CAD [7,8]. As a dynamic marker while one experiences different loads, HRV appears to be sensitive and responsive to acute stress, including exercise [7,9]. 

However, the diagnostic potential of exercise-related HRV was still controversial in diagnosing CAD. Bailon et al. identified that HRV indices corrected by mean heart rate (HR) and respiratory frequency could improve the accuracy of EST, ranging from 76% to 95% [10]. Yet, some previous findings reported that the values of HRV indices alone or corrected with HR were insufficient in distinguishing CAD detection [11,12]. Therefore, this study aimed to evaluate the correlation between short-term HRV and myocardial ischemia during EST, including the acceleration, maximum, and recovery stages of heart rate (HR).

## 2. Materials and Methods

### 2.1. Study Population

After excluding ECG abnormalities including LBBB, paced rhythm, Wolff-Parkinson-White syndrome, ≥0.1 mV ST-segment depression on resting ECG, or treatment with digitalis, 54 patients who were performing EST were enrolled at Hsinchu MacKay Memorial Hospital. A coronary imaging test was performed if EST was positive. Of 35 patients that underwent coronary imaging test, 10 patients (age: 54.7 ± 10.9 ys, male/female: 8/2) had significant CAD (sCAD) (stenosis ≥ 50%) and 25 patients (age: 49.7 ± 10.4 ys, male/female: 16/9) were insignificant CAD (iCAD) (stenosis < 50%). The remaining 19 patients (age: 48.0 ± 10.4 ys, male/female: 13/6) with negative EST were defined as having relative health control (RHC). This study was approved by the MacKay Hospital Institutional Review Board (17MMHIS004e). 

### 2.2. Exercise Stress Testing (EST)

The EST follows the Bruce protocol. It consists of seven stages, with a gradual incremental increase in the speed and gradient of the treadmill during the EST. The predicted peak heart rate was calculated as 220-age. Individuals were encouraged to exercise until they experienced limiting symptoms, even if 85% of the maximum predicted heart rate was achieved [13]. Criteria for exercise termination were physical exhaustion or maximum heart rate greater than the age-predicted maximum. During each exercise stage and recovery stage, symptoms, blood pressure, heart rate, and exercise workload in metabolic equivalents (METS) were recorded. Each stage lasted three minutes to allow the patient to habituate to increasing speed and gradient before advancing to the next stage. Following peak exercise, individuals walked for a two-minute cool-down period at 1.5 mph and 2.5% grade. The exercise tests were performed, analyzed, and reported with a standard protocol utilizing a computerized database. The results of the EST were reconfirmed by two cardiologists. 

### 2.3. RR Intervals Extraction

All patients underwent short-term electrocardiographic recording, which was performed using the “Medilog” Digital Holter Recorder (Medilog Darwin v2.3 AR4 plus) during EST. RR intervals were extracted from subjects’ ECG during EST. Ectopic and other artifactual beats within the RR interval series were corrected using the interpolation method [14]. Drift in RR interval series was minimized using empirical mode decomposition. After the preprocessing, nine segments of the 1 min RR interval series were extracted at different stages before and after the exercise. The 9 stages include the rest period before exercise (Res), the first minute before maximal heart rate (Pre), the first minute when the maximum heart rate was reached (Max), and every 1 min of 6 min in the post-exercise recovery phase (R1~R6). 

### 2.4. Analysis of Heart Rate Variability

HRV analysis is suitable for evaluating the short-term autonomic regulation of heart rate under physiologically stable conditions [15]. HRV analysis was applied to the nine one-minute RR interval series of all 54 patients. The HRV metric includes indices from time and frequency domain analysis and non-linear Poincaré plot analysis.

Time domain HRV indices include the root mean square of successive RR differences (RMSSD), the standard deviation of all normal to normal intervals (SDNN), the mean value of the RR interval series (MRRI), and the mean of heart rate (MHR). SDNN reproduces the total variability, and RMSSD reflects parasympathetic activity [7]. Frequency domain HRV indices include the total power from 0.0033 to 0.4 Hz (TP), the very low-frequency power from 0.0033 to 0.04 Hz (VLF), the low-frequency power from 0.04 to 0.15 Hz (LF), the high-frequency power from 0.15 to 0.4 Hz (HF), the ratio of low-frequency power to high-frequency power (LF/HF), the normalized low-frequency power (LF (nu) = LF/(LF + HF)), and the normalized high-frequency power (HF (nu) = HF/(LF + HF)). Index LF referring to the modulation of the RR interval changes corresponds to the sympathetic and parasympathetic activity together. HF modulation of the RR interval changes is primarily regulated by the parasympathetic nerve through the innervations of the heart [7]. Non-linear indices of Poincaré plot analysis include the standard deviation in the short axis direction (SD1), the standard deviation in the long axis direction (SD2), and SD1/SD2. SD1 is the fast beat-to-beat variability in the RR intervals, while SD2 describes the longer-term variability. SD1 reflects mainly the parasympathetic input to the heart, while SD2 reflects the sympathetic and parasympathetic contributions to the heart [7,16].

Further, for each HRV index of a patient, the slope of the linear regression from Max to R6 stages was calculated to quantify the recovery ability of the patient.

### 2.5. Statistical Methods

The HRV data were presented as mean  ±  standard deviation. The degree of difference for each HRV index between any two groups was performed in terms of *p* value using the Wilcoxon rank-sum test (which is suitable for small sample comparison based on non-parametric statistics). The statistical significance was set at *p*  <  0.05. All data analyses were performed using Python’s open-source statistical package, Pingouin v0.3.3. [17]

## 3. Results

A total of 54 patients were enrolled in the study. They were divided into three groups: RHC (n = 19; mean age: 51.9 ± 8.8 years; male/female, 13/6), sCAD (n = 10; mean age: 54.7 ± 10.0 years; male/female, 8/2), and iCAD (n = 25; mean age: 53.4 ± 8.9 years; male/female, 16/9). There were no significant differences in sex and age among the groups. 

### 3.1. Comparison of HRV between RHC and sCAD Groups in Individual EST Stages

**Figure 1:**
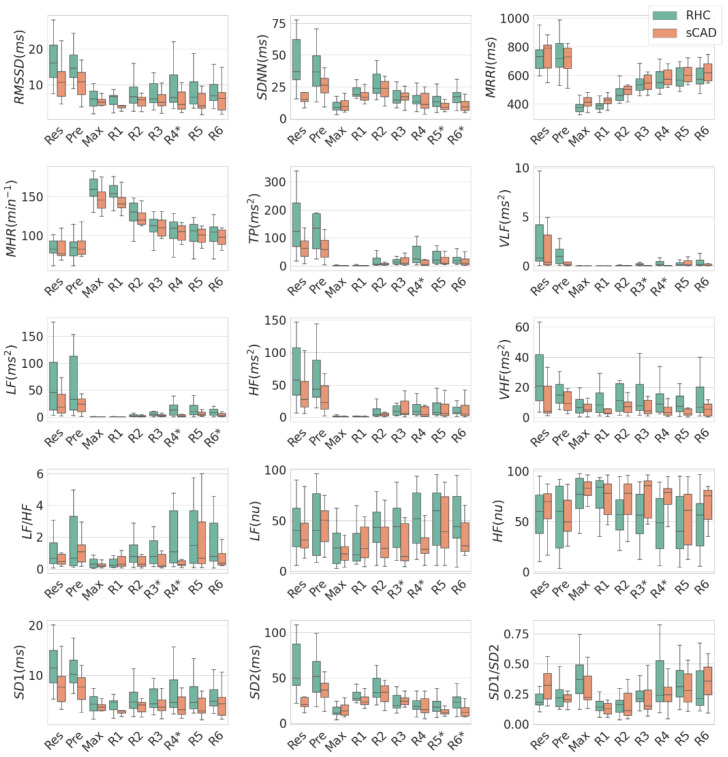
The box plot of time- and frequency-domain HRV indices of the sCAD and the RHC groups at rest, maximum heart rate, and recovery stage (R1~R6). The symbol * indicates a significant difference at the 0.05 level (*p* < 0.05).

Figure 1 compares the HRV indices between the RHC and the sCAD groups at all 9 stages. From Res to Max stages, most HRV indices decreased except for the MHR. From R3 to R6 stages, most HRV indices of the RHC group, except for the HF (nu), recovered faster than those of the sCAD group. Note that the mean value of LF in Max stage was nearly negligible. This indicates that the values of LF in Max stage were nearly the same for all subjects in both groups. This is a unique feature to be applied later.

Table 1 shows a comparison of HRV indices in different recovery stages between the RHC and the sCAD groups. In the R1 and R2 stages, there was no significant difference between the two groups. In the R3 stage, the VLF, LF, and LF (nu) were lower in the sCAD group than in the RHC group. In the R4 stage, the VLF, LF, LF/HF, TP, LF (nu), rMSSD, and SD1 were lower in the sCAD group. In the R5 stage, the SDNN and SD2 were lower in the sCAD group. In the R6 stage, the LF, SDNN, and SD2 were lower in the sCAD group. Among the HRV indices listed in Table 1, the LF in R4 (35.77 ± 64.06 vs. 3.39 ± 4.3 ms^2^) exhibited the best performance (*p* < 0.01), which is labeled in bold in Table 1.

### 3.2. Comparison of HRV between RHC and sCAD Groups in Individual EST Stages with Respect to Max Stage

**Figure 2:**
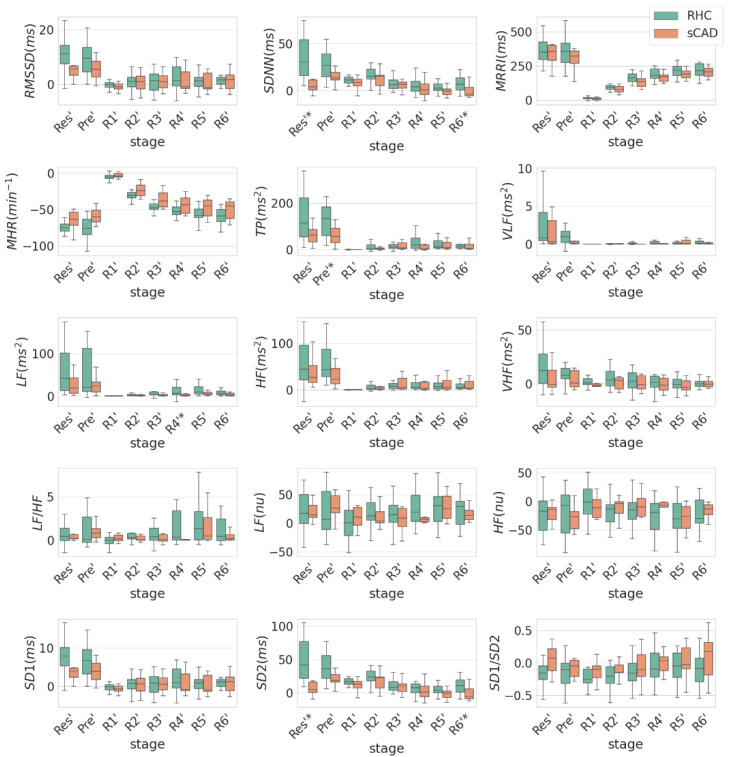
The box plot of the difference in the value of each HRV index between each stage and the Max stage. Each stage has a prime labeling. The symbol * indicates a significant difference at the 0.05 level (*p* < 0.05).

Figure 2 shows the difference in the value of each HRV index between each stage and the Max stage. From the Res to Max stage, the relative reductions of the HRV indices were significantly higher in the RHC groups than in the sCAD group, especially for SDNN, SD2 and total power. From the Max to R4 stage, the relative increase of LF was significantly higher in the RHC group than in the sCAD group. From the Max to R6 stage, the relative increase of HRV was significantly higher in RHC groups, especially for SDNN and SD2.

Table 2 shows a comparison of the HRV indices between the RHC and sCAD groups in individual EST stages with respect to Max stage. Only particular stages are listed in which the HRV indices between the two groups are significantly different. For the purpose of comparison, LF, HF, and LF/HF are also listed. Yet, HF and LF/HF showed no statistical difference between the two groups. Among the HRV indices listed in Table 2, the SDNN (34.45 ± 21.28 vs. 11.08 ± 17.89 ms) and SD2 (48.3 ± 30.04 vs. 14.89 ± 25.42 ms) in Res with respect to Max exhibited the best performance (*p* < 0.01).

**Figure 3:**
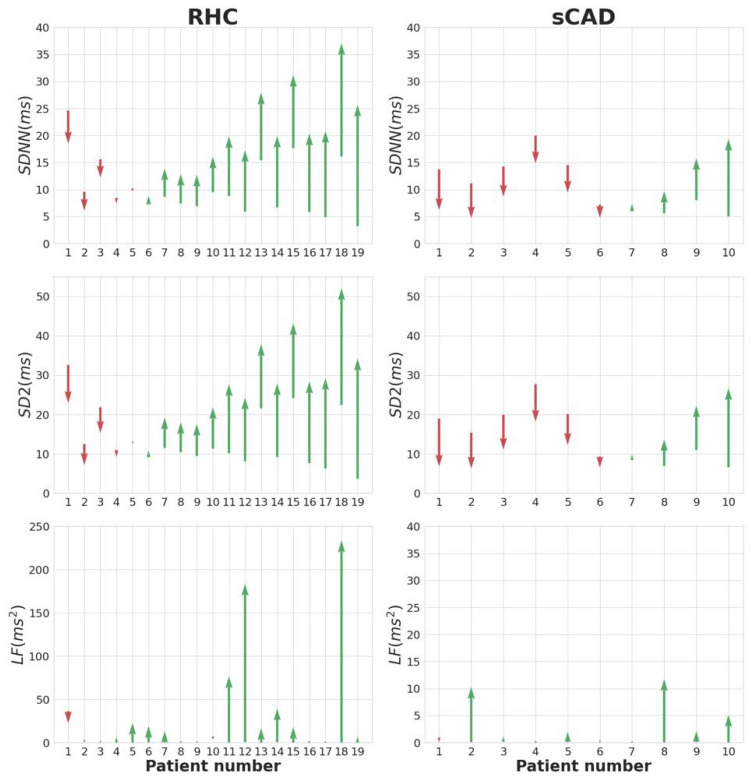
The HRV trend of each individual in the RHC and sCAD groups. The starting point of each arrow is the corresponding HRV value at the Max stage. The ending points of the arrows in the SDNN, SD2, and LF panels are the corresponding HRV values at the R6, R6, and R4 stages, respectively, as specified in Table 2.

According to Table 2, the recoveries of SDNN from Max to R6, SD2 from Max to R6, and LF from Max to R4 in the RHC group were significantly higher than those in the sCAD group. Figure 3 illustrates the corresponding recovery amount of SDNN, SD2, and LF of each patient in the RHC and the sCAD groups. Green and red arrows indicate an increase and decrease in HRV values, respectively. In the RHC group, most patients had a significant increase in SDNN, SD2, and LF indices. Furthermore, the arrow colors of LF tend to be more consistent for all patients than those of SDNN or SD2 in each group.

### 3.3. The Recovery Slope of HRV of the RHC, iCAD, and sCAD Groups

To quantify the recovery intensity of each HRV index, we calculated the slope of the linear regression from the Max to the R6 stages of the index for each subject in the RHC, sCAD, and iCAD groups. Table 3 shows the population characteristics of the 54 patients and their recovery slope (mean ± standard error) of each HRV index with a *p* value between any two groups during the recovery phase. For comparison, the slopes of LF, SDNN, and SD2 were significantly lower in the sCAD group than in the RHC group. Among the three indices, the slope of LF exhibited the best performance (3.93 ± 5.95 vs. 0.85 ± 0.68 ms^2^/min, *p* = 0.02) to separate the sCAD group from the RHC group. On the contrary, although the slope of LF still exhibited the best performance (3.82 ± 3.82 vs. 0.85 ± 0.68 ms^2^/min, *p* = 0.06) between the iCAD and sCAD groups, there was no significant difference (*p* = 0.06). Similarly, there was also no significant difference (*p* = 0.57) between the iCAD and RHC groups.

**Figure 4:**
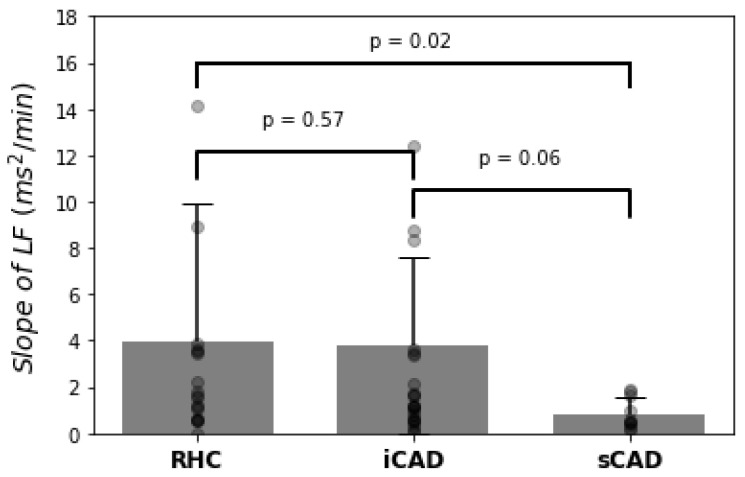
The slope of the linear regression from the Max to the R6 stages of LF for each of the 54 patients in the RHC, sCAD, and iCAD groups, individually. Bars indicate mean ± standard error. Each circle represents one individual.

Figure 4 shows the slope of the linear regression from the Max to the R6 stages of LF for each of the 54 patients in the RHC, sCAD, and iCAD groups, individually. Obviously, the slope of LF could be used for differentiating both the RHC and iCAD patients from the sCAD ones.

## 4. Discussion

EST increases the load of the heart via physical exercise and is a risk assessment method for screening asymptomatic CAD patients [1]. However, the accuracy of EST was lower for diagnosing CAD and could be influenced by the patient’s gender, age, and underlying disease [3,5]. A meta-analytic review in 2012 reported a positive likelihood ratio of 3.57 and a negative likelihood ratio of 0.34 for EST [3]. Therefore, EST is only recommended for risk assessment rather than for diagnosing CAD, due to its poor diagnostic accuracy [4]. Although invasive coronary angiography (CAG) is the gold standard for detecting CAD, it may be accompanied by a possible risk of arrhythmia, myocardial infarction, stroke, and some artery injury [1]. Therefore, adding a non-invasive imaging study, energy defect of Hilbert–Huang transform (HHT), or HRV with EST could help to increase the diagnostic accuracy of CAD [4,18]. 

Physical exercise is associated with parasympathetic withdrawal and sympathetic increase, resulting in a heart rate increase. The analysis of HRV has been used to predict cardiovascular mortality, but is not usually used in predicting CAD due to inconsistent results [12,19,20,21]. After exercise cessation, HR and HRV demonstrate a time-dependent recovery and return to pre-exercise levels. The initial increasing HR during exercise is due to parasympathetic withdrawal, and recovery of the HR immediately after exercise is mediated by parasympathetic reactivation in response to the activity in arterial baroreceptors [22,23]. The heart rate recovery (HRR), especially in the first 30 s after exercise, reflects the balance of reactivating parasympathetic drive and withdrawal of sympathetic drive [19]. The imbalance of autonomic function, especially the delayed reactivation of the parasympathetic nervous system, plays an important role in HHR and predicts cardiovascular mortality [19,20,21,24]. The last phase of HRR has been attributed to parasympathetic reactivation [25]. Slow HRR of the early post-exercise period reflects the autonomic imbalance and could predict all-cause mortality, sudden death, or silent ischemia [19,22,23] independent of exercise workload, myocardial perfusion defects, or HR changes during exercise [26]. However, the possible mechanisms mediating post-exercise cardio-deceleration are not well known. They could be related to autonomic denervation of the myocardium, a higher pain threshold during EST, higher β-endorphin, or anti-inflammatory cytokines [22,23]. Slow HRR could predict microvascular or macrovascular myocardial ischemia, but the relation between HRR and the severity of coronary stenosis is still uncertain [22]. HRR in the supine position shortly after exercise may have a negative predictive value for CAD detection [27]. In our study, the sCAD group had a lower maximum HR and slower HRR during the R1 and R2 stage compared with the RHC group. During the recovery stage of EST in the sCAD group, a slower phase of cardio-deceleration was observed, likely mediated by both prolonged parasympathetic reactivation and sympathetic withdrawal. 

The complexity of HRV is moderated by the dynamic interaction between the sympathetic and parasympathetic nervous systems [16]. Compared with HRR, the complexity of HRV is not yet fully understood in supposed correspondence with different HRV powers [28]. By applying these frequency range differences in HRV analysis, the individual contribution of parasympathetic and sympathetic systems could be identified by HRV analysis. Typically, LF-nu increases during low-moderate intensity exercise and decreases during higher-intensity exercise, while HF-nu demonstrates the opposite response [25]. High-intensity exercise training can chronically lead to a shift from vagal to sympathetic cardiac modulation, and the progressive sympathetic predominance at peak training load may predict performance during exercise [7]. A transition from parasympathetic to sympathetic predominance in cardiovascular autonomic modulation could be influenced by different intensity during exercise, duration of exercise, and acute post-exercise recovery [7]. Regular exercise would activate the parasympathetic activity and has been shown to increase resting HRV [28]. However, the role of HRV recovery after acute EST still remains unclear. Furthermore, the variation of the HF component is largely contributed to by respiratory sinus arrhythmia (RSA). Vagal-mediated RSA may fluctuate greatly with cardio-acceleration during inspiration and cardio-deceleration during expiration [7]. As the workload of the EST increases, decreasing HRV is noted when respiratory frequency increases [7]. Some patients may experience irregular respiratory rate during angina, thereby rendering difficult the proper interpretation of HRV data [10,12]. 

In this study, patients with significant CAD may have undergone a lower intensity or shorter duration of exercise, for they usually complained of angina earlier than the expected intensity or duration was reached. This tended to influence their heart rates and the corresponding HRV inconsistently and individually. In the Max stage, although the heart rates and exercise intensities were different among patients, the values of some HRV indices such as LF were found to remain the same, nearly negligible, for all subjects in the RHC and the sCAD groups, as shown in Figure 1. Thus, monitoring the HRV recovery after the Max stage may be an alternative method for assessing heart load recovery. 

Among HRV indices, the HF power may fluctuate substantially, and LF/HF also demonstrates inconsistent responses to different intensities of exercise [25]. As they have insufficient power in distinguishing coronary stenosis or vessel number, LF, HF, or VLF components of HRV have been neglected in recent HRV studies [11,29]. The LF of HRV was significantly lower in patients with CAD [11] and could be another index for distinguishing myocardial ischemia. In a previous study, myocardial ischemia induced sympathetic reflex and delayed recovery of LF/HF during the early recovery period of EST [30]. 

In this study, the delayed reactivating parasympathetic tone of the sCAD group was observed in the HF index during the R3 and R4 stages. Withdrawal of sympathetic tone with prolonged lower LF index was found in patients with significant CAD during the whole recovery stage. Reduced HRV and HRR were risk factors of CAD after multi-variable regression analysis [11,31]. However, we found that delayed HRV recovery, especially in regards to the low recovery slope of LF, occurred in patients with significant CAD. In the recovery stage of EST, the recovery amounts of LF were significantly lower in the sCAD group than in the RHC group. Additionally, the recovery slope of LF power was also significantly lower in the sCAD group. Therefore, post-exercise HRV, especially in regards to the recovery slope of LF, could be an important tool for investigating autonomic imbalance in patients with significant CAD. Although there was no significant difference between the sCAD and iCAD groups, the mean values of the recovery slopes of LF in the sCAD, iCAD, and RHC groups are relatively small, medium, and large, respectively, as expected. The result implies that the slope of LF can be partially applied to reflect the severity of coronary stenosis. In future studies, combining machine learning techniques with multiple HRV indices may increase the accuracy in quantifying the severity of coronary stenosis.

This study possessed the limitations of being performed at a single institution and in a small patient population. In addition, the gender imbalance of participants may affect the results [32]. The difference in exercise parameters between the three groups is another limitation of this retrospective data. However, the autonomic imbalance was more significant during the post-exercise period irrespective of different exercise stress influences. In the future, the relation between decreasing sympathetic activity and CAD should be assessed in a larger number of subjects with pathophysiologic data. In conclusion, although it is possible that these limitations influenced the results, we conclude that, in this preliminary study, the decreasing sympathetic activity, especially as it relates to the recovery slope of LF, is useful for easily detecting myocardial ischemia in high-risk patients and could increase the accuracy of EST without unnecessary invasive treatments.

## 5. Conclusions

The main finding of the present study indicated that short-term HRV could reflect the cardiovascular autonomic imbalance in patients with significant CAD during EST. Moreover, the impaired response of HRV after exercise, especially the prolonged withdrawal of sympathetic tone, was found. A relatively slow recovery slope of LF after exercise could be an additional marker for diagnosing myocardial ischemia.

## Figures and Tables

**Table 1:** A comparison of HRV indices in different recovery stages between the RHC and the sCAD groups. Only particular recovery stages are listed in which the HRV indices between the two groups are significantly different. The LF in R4 in bold exhibited the best performance (*p* < 0.01).

	Units	Stage	RHC (n = 19)	sCAD (n = 10)	*p*-Value
**VLF**	ms^2^	R3	0.23 ± 0.4	0.04 ± 0.08	0.03
**LF/HF**		R3	1.57 ± 1.97	0.44 ± 0.46	0.04
**LF (nu)**		R3	45.02 ± 25.32	25.02 ± 20.3	0.04
**HF (nu)**		R3	54.98 ± 25.32	74.98 ± 20.3	0.04
**VLF**	ms^2^	R4	0.85 ± 1.98	0.04 ± 0.06	0.01
**LF/HF**		R4	3.41 ± 4.87	0.54 ± 0.67	0.02
**LF**	ms^2^	**R4**	**35.77 ± 64.06**	**3.39 ± 4.3**	**<0.01**
**TP**	ms^2^	R4	54.11 ± 69.41	16.31 ± 22.09	0.02
**LF (nu)**		R4	52.18 ± 28.7	27.36 ± 20.32	0.02
**HF (nu)**		R4	47.82 ± 28.7	72.64 ± 20.32	0.02
**RMSSD**	ms	R4	8.67 ± 4.89	6.13 ± 4.54	0.05
**SD1**	ms	R4	6.16 ± 3.48	4.35 ± 3.23	0.05
**SDNN**	ms	R5	14.12 ± 6.07	9.61 ± 3.28	0.05
**SD2**	ms	R5	18.92 ± 8.37	12.32 ± 3.67	0.03
**LF**	ms^2^	R6	13.6 ± 18.86	3.67 ± 3.38	0.02
**SDNN**	ms	R6	17.8 ± 8.27	10.09 ± 4.98	0.01
**SD2**	ms	R6	24.23 ± 11.78	13.34 ± 6.99	0.01

**Table 2:** A comparison of HRV indices between RHC and sCAD groups in individual EST stages with respect to Max stage. Only particular stages are listed in which the HRV indices between the two groups are significantly different. For the purpose of comparison, LF, HF, and LF/HF are also listed, which showed no statistical difference between the two groups. The SDNN and SD2 in Res with respect to Max, in bold, exhibited the best performance (*p* < 0.01).

HRV	Units	Stage Level	RHC (n = 19)	sCAD (n = 10)	*p*-Value
**SDNN**	ms	**Res—Max**	**34.45 ± 21.28**	**11.08 ± 17.89**	**<0.01**
**SD2**	ms	**Res—Max**	**48.3 ± 30.04**	**14.89 ± 25.42**	**<0.01**
**Total Power**	ms^2^	Pre—Max	182.83 ± 203.7	61.16 ± 42.45	0.03
**LF**	ms^2^	R4—Max	33.35 ± 65.15	3.23 ± 4.38	0.04
**HF**	ms^2^	R4—Max	9.98 ± 25.01	12.14 ± 17.93	0.54
**LF/HF**		R4—Max	2.98 ± 4.81	0.31 ± 0.62	0.15
**SDNN**	ms	R6—Max	7.67 ± 8.34	−0.46 ± 7.21	0.02
**SD2**	ms	R6—Max	10.79 ± 11.96	−1.07 ± 10.75	0.02

**Table 3:** Population characteristics and the recovery slope (means ± standard deviation) of each HRV index with a *p* value between any two groups during the recovery phase. Bold highlights the best performances. The symbol * indicates a significant difference at the 0.05 level (*p* < 0.05).

		RHC (n = 19)	iCAD (n = 25)	sCAD (n = 10)	*p*-Value		
					RHC vs. iCAD	RHC vs. sCAD	iCAD vs. sCAD
**Age**	ys	48.0 ± 10.4	49.7 ± 10.4	54.7 ± 10.9	0.96	0.17	0.21
**Gender** **(male/female)**		13/6	16/9	8/2			
**HRV** **indices**	Units						
**RMSSD**	ms/min	0.39 ± 0.86	0.41 ± 0.41	0.28 ± 1.05	0.8	0.66	0.57
**SDNN**	ms/min	−0.15 ± 1.13	−0.57 ± −0.57	−0.96 ± 0.88	**0.17**	0.04 *	0.5
**MRRI**	ms/min	42.0 ± 13.58	43.01 ± 43.01	39.52 ± 10.41	0.71	0.7	0.32
**MHR**	min^−2^	−10.68 ± 1.94	−10.0 ± −10.0	−9.25 ± 2.33	0.24	0.12	0.3
**TP**	ms^2^/min	5.73 ± 6.96	6.85 ± 6.85	3.61 ± 4.56	0.76	0.11	0.12
**VLF**	ms^2^/min	0.08 ± 0.11	0.07 ± 0.07	0.04 ± 0.05	0.92	0.6	0.2
**LF**	ms^2^/min	3.93 ± 5.95	3.82 ± 3.82	0.85 ± 0.68	0.57	0.02 *	**0.06**
**HF**	ms^2^/min	1.72 ± 2.66	2.96 ± 2.96	2.72 ± 3.93	0.39	0.8	0.39
**VHF**	ms^2^/min	0.46 ± 2.95	0.56 ± 0.56	0.5 ± 4.0	0.89	0.87	0.65
**LF/HF**	min^−1^	0.52 ± 0.89	0.41 ± 0.41	0.16 ± 0.22	0.59	0.35	0.39
**LF (nu)**	min^−1^	4.87 ± 5.03	4.24 ± 4.24	2.96 ± 3.31	0.76	0.32	0.35
**HF (nu)**	min^−1^	−4.87 ± 5.03	−4.24 ± −4.24	−2.96 ± 3.31	0.76	0.32	0.35
**SD1**	ms/min	0.28 ± 0.62	0.29 ± 0.29	0.2 ± 0.75	0.79	0.66	0.57
**SD2**	ms/min	−0.29 ± 1.66	−0.91 ± −0.91	−1.49 ± 1.33	0.18	0.05 *	0.37
**SD1/SD2**	min^−1^	0.01 ± 0.03	0.02 ± 0.02	0.03 ± 0.06	0.64	0.54	0.6

## Data Availability

The anonymized data are available from the author upon reasonable request.

## Abbreviations

EST: Exercise stress testing; CAD: Coronary artery disease; HRV: Heart rate variability; RHC: Relative health control; iCAD: Insignificant CAD; sCAD: Significant CAD; ECG: Electrocardiogram; ESC: European Society of Cardiology; CT: Computed tomography; METS: Metabolic equivalents; Res: Rest period before exercise; Pre: The first minute before maximal heart rate; Max: The first minute when the maximum heart rate was reached; R1~R6: Every 1 min of 6 min in the post-exercise recovery phase; RMSSD: Root mean square of successive RR differences; SDNN: Standard deviation of all normal to normal intervals; MRRI: The mean value of the RR interval series; MHR: Mean of heart rate; TP: Total power from 0.0033 to 0.4 Hz; VLF: Very low-frequency power from 0.0033 to 0.04 Hz; LF: Low-frequency power from 0.04 to 0.15 Hz; HF: High-frequency power from 0.15 to 0.4 Hz; LF/HF: The ratio of low-frequency power to high-frequency power; LF (nu): Normalized low-frequency power (LFnu = LF/(LF + HF)); HF (nu): Normalized high-frequency power (HFnu = HF/(LF + HF)); SD1: Standard deviation in the short axis direction of Poincaré plot analysis; SD2: Standard deviation in the long axis direction of Poincaré plot analysis; CAG: Coronary angiography; HHT: Hilbert–Huang transform; HRR: Heart rate recovery.
